# Benchmark dataset for the Asymmetric and Clustered Vehicle Routing Problem with Simultaneous Pickup and Deliveries, Variable Costs and Forbidden Paths

**DOI:** 10.1016/j.dib.2020.105142

**Published:** 2020-01-28

**Authors:** Eneko Osaba

**Affiliations:** Tecnalia Research & Innovation, Parque Cientifico y Tecnologico de Bizkaia, Geldo Auzoa, 700 Building, 48160, Derio, Vizcaya, Spain

**Keywords:** Optimization, Vehicle routing problem, Multi-attribute routing problem, Newspaper delivery, Operation research, Traveling salesman problem, Combinatorial optimization

## Abstract

In this paper, the benchmark dataset for the Asymmetric and Clustered Vehicle Routing Problem with Simultaneous Pickup and Deliveries, Variable Costs and Forbidden Paths is presented (AC-VRP-SPDVCFP). This problem is a specific multi-attribute variant of the well-known Vehicle Routing Problem, and it has been originally built for modelling and solving a real-world newspaper distribution problem with recycling policies. The whole benchmark is composed by 15 instances comprised by 50–100 nodes. For the design of this dataset, real geographical positions have been used, located in the province of Bizkaia, Spain. A deep description of the benchmark is provided in this paper, aiming at extending the details and experimentation given in the paper *A discrete firefly algorithm to solve a rich vehicle routing problem modelling a newspaper distribution system with recycling policy* (Osaba et al.) [1]. The dataset is publicly available for its use and modification.

**Table:** Specifications Table

Subject	Artificial Intelligence
Specific subject area	Control and Optimization, Discrete Mathematics and Optimization
Type of data	Table, Image, Text Files
How data were acquired	All instances have been artificially generated in a laboratory environment. For the positions of all the 100 clients, real-world locations have been considered using the common geographic coordinate system. For enhancing the understandability of the benchmark, a map has been generated with the location of both clients and depot, using Open Street Maps technology, via uMap tool (https://umap.openstreetmap.fr.).
Data format	Raw
Parameters for data collection	The data has been collected in a laboratory environment. All the data regarding travel costs, pick-up and delivery demands, vehicle capacities and forbidden paths have been synthetically generated using different mathematical formulas, introduced in Ref. [1].
Description of data collection	All the information regarding locations has been obtained using Open Street Map environment (https://www.openstreetmap.org) and Geographic Coordinate System.
Data source location	All the information corresponds to real client locations, placed in Biscay, Spain. All locations are placed in rectangular a frame with 43.360222, −3.103410 as north-west limit, and 43.238985, −2.877617 as south-east limit.
Data accessibility	All the data is available in a public repository Repository name: Joint Research Lab Software Repository (http://jrlab.science/software) Direct URL to data: (https://github.com/Maldini32/AC-VRP-SPDVCFP/)
Related research article	This benchmark is related with the following research paper: Osaba, E., Yang, X. S., Diaz, F., Onieva, E., Masegosa, A. D., & Perallos, A. (2017). A discrete firefly algorithm to solve a rich vehicle routing problem modelling a newspaper distribution system with recycling policy. Soft Computing, 21 (18), 5295–5308. DOI: https://doi.org/10.1007/s00500-016-2114-1

**Table:** 

**Value of the Data** • This benchmark includes 15 realistic dataset which are based on the logistic distribution of newspapers with recycling policy. Both size and nature of the instances are consistent with newspaper delivery problems found in practice. Thus, datasets provided are useful for being used for assessing the performance of solvers developed with practical purposes. • Researchers can employ the presented datasets for benchmarking purposes, and for the comparison of different existing and newly developed solvers, including exact methods, heuristics and meta-heuristics. • Researchers dealing tactical and strategic newspaper delivery problems can be benefited from this benchmark, since large datasets with large number of clients can be employed for measuring modelled solvers for this kind of problems, with different characteristics such as recycling policies, forbidden roads or variable travel times. • Datasets are flexible enough for being extended to other real-world oriented Rich Vehicle Routing Problems, extending in this way their use to other variants [2], and allowing the obtaining of further insights and the development of additional experiments. • Features of the realistic datasets suppose an additional value, allowing the comparison with other real-world newspaper delivery systems for identifying common features that can directly influence the performance of developed solvers.

## 1 Data description

The benchmark accompanying this article consists on 15 different dataset instances for the Asymmetric and Clustered Vehicle Routing Problem with Simultaneous Pickup and Deliveries, Variable Costs and Forbidden Paths (AC-VRP-SPDVCFP). This problem is a rich variant [3] of well-known Vehicle Routing Problem and it represents a newspaper delivery problem with recycling policies. These are the main characteristics of the problem:

• *Asymmetry* [4]: This feature implies that the cost of traveling from one client to another one is different from its reverse trip. Furthermore, every relation between two different nodes is affected by this asymmetry.
• *Clustered* [5]: All nodes that comprise the whole scenario are grouped into different sets or clusters. Additionally, a hard restriction is involved with this condition: if a vehicle visits a client, it must compulsory visit all customers belonging the same cluster in the same route. Otherwise, if the vehicle has not the capacity enough to serve all clients in a set, it cannot visit any node of the cluster.
• *Variable Travel Times* [6]: A working schedule has been set for this problem. Specifically, this working schedule starts at 6:00 and finishes at 15:00. Moreover, within this time-window, two different periods have been established: *peak hours* and *valley hours*. First ones correspond to the period between 8:00 and 10:00. All services conducted within this time window involve a higher traveling cost. Remaining hours are deemed as *valley hours*.
• *Simultaneous Pickup and Delivery* [7]: This feature involves the existence of two types of nodes. First ones are points in which newspapers are delivered. On the other hand, the second ones correspond to the nodes in which newspapers are gathered for being transported to the depot, for their posterior recycling.
• *Forbidden Paths* [8]: This restriction implies the existence of a variable number of streets and paths that cannot be traversed by the vehicles for building the solution of the problem.

All datasets are freely available in Ref. [9], and also in Mendeley. The whole benchmark is composed by 15 different instances, a descriptive map with the geographical distribution of the clients, an informative text file with all the latitudes and longitudes of the customers, and an indicative XML parser for facilitate the reading of the instances. In addition, all datasets are called *Osaba_X_Y_Z*, where *X* is the number of customers, *Y* the distribution of the clusters, and *Z* the distribution of the clients. All these details are deeply explained in the following section.

**Fig. 1:**
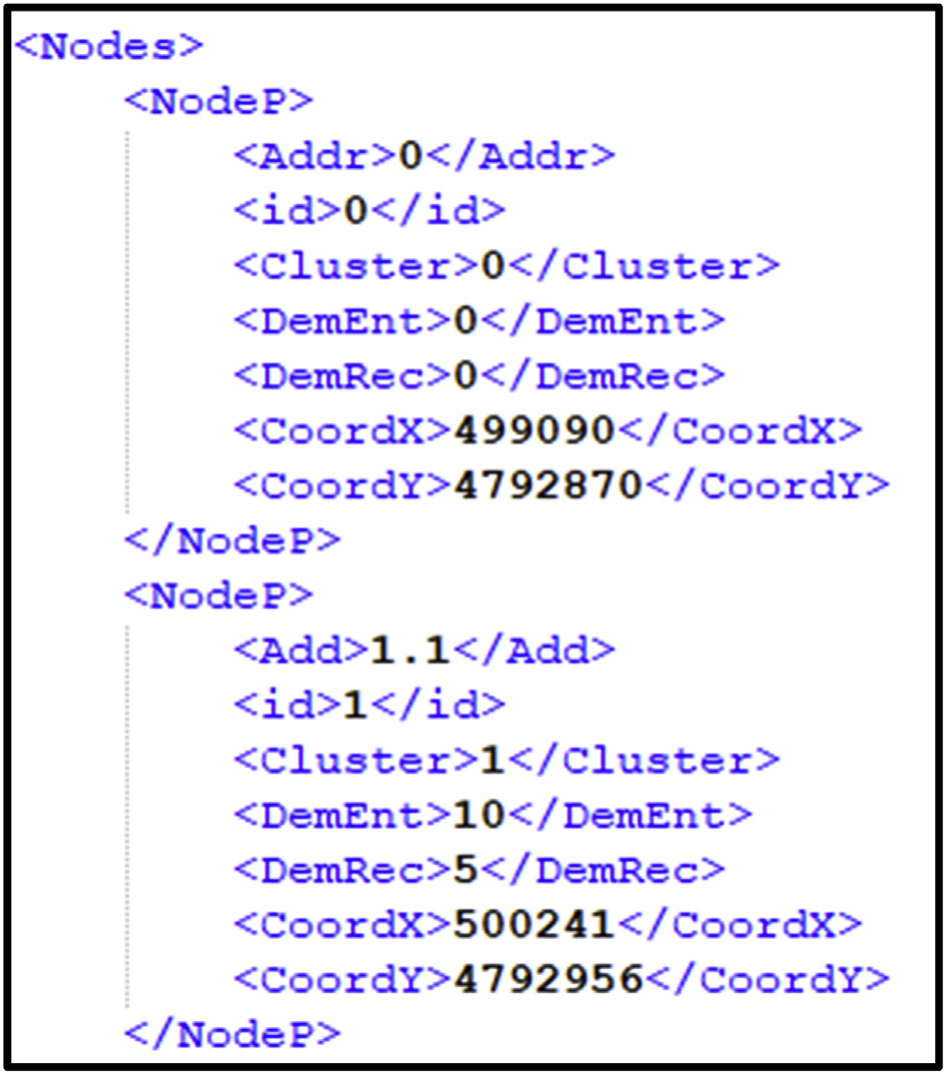
Brief excerpt of an AC-VRP-SPDVCFP benchmark instance.

In the following Fig. 1, a brief excerpt of a dataset is shown. All instances are XML files, containing the list of all clients that comprise the dataset. Within the list, the information of the depot is also available. As can be observed, for each client the following information is provided: address, identification, cluster, delivery demand, pickup demand, coordinate *X* and coordinate *Y*.

## 2 Experimental design, materials, and methods

**Fig. 2:**
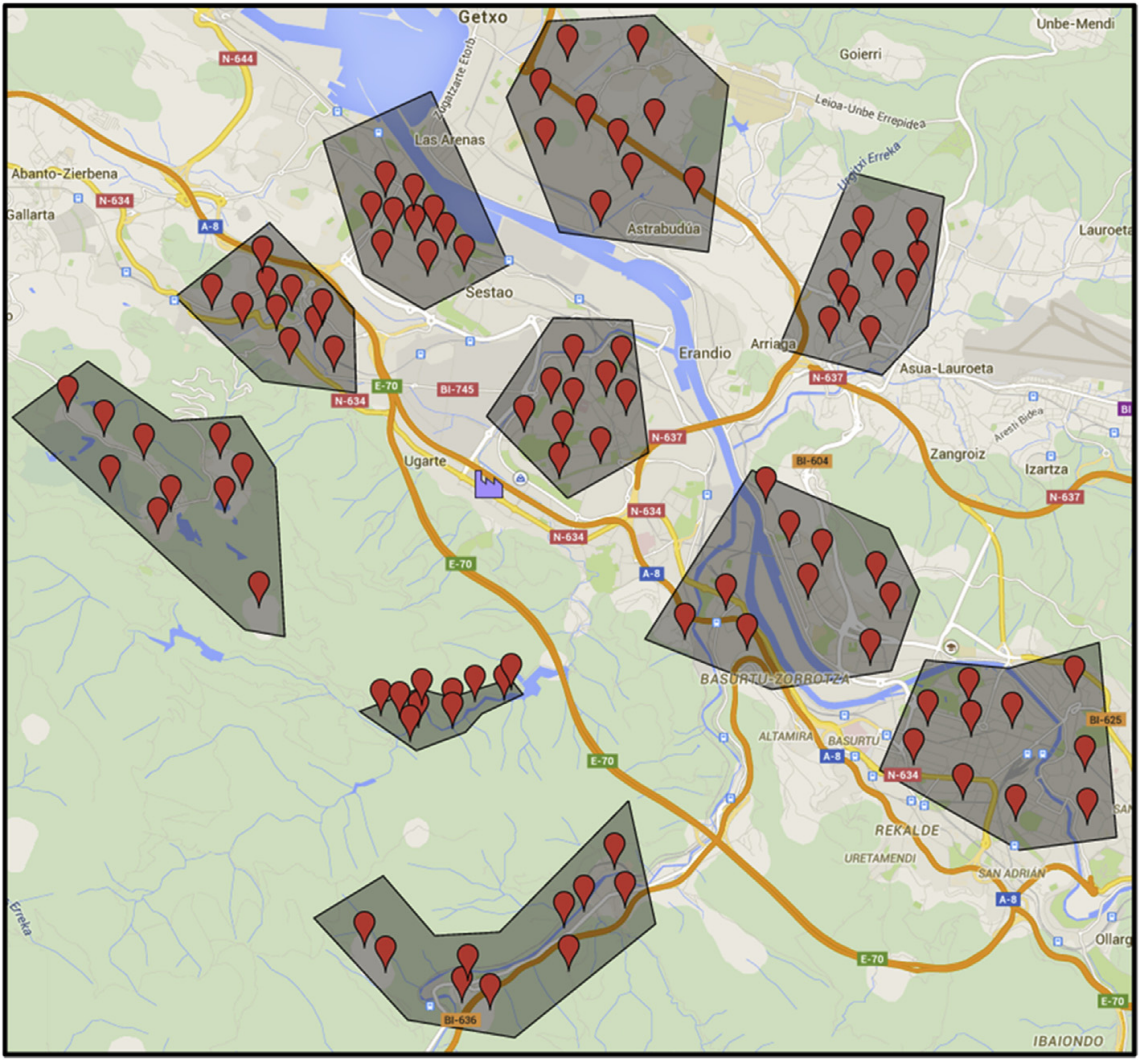
Geographical locations of the depot, customers and clusters around the province of Bizkaia. Source: Google Maps.

In this section, the process followed to generate the whole benchmark is detailed. For enhancing the contextualization of the datasets, the Geographical locations of the depot, customers and clusters around the province of Bizkaia are illustrated in Fig. 2. Further information can be found in Ref. [1].

As has been introduced above, the benchmark is composed by 15 instances. Moreover, these datasets are composed by 50–100 nodes. Each node represents a client, placed in specific geographical location. Furthermore, the maximum number of clusters has been established in 10, existing also datasets with 5, and 8 cluster.

For the generation of these clusters, they have been build using the order of appearance. This means that first ten customers comprise the first clusters, second ten clients build the second cluster, and so on. It is also important to highlight that all the clusters that compose a dataset contain the same amount of nodes. Regarding the assignment of both delivery and pick-up demands, the following formula has been used:

$$d_{i}=10,\;p_{i}=5,\;\forall i\;\in \left\{1,5,9,\ldots 97\right\}$$

$$d_{i}=10,\;p_{i}=0,\;\forall i\;\in \left\{2,6,10,\ldots 98\right\}$$

$$d_{i}=5,\;p_{i}=3,\;\forall i\;\in \left\{3,7,11,\ldots 99\right\}$$

$$d_{i}=5,\;p_{i}=0,\;\forall i\;\in \left\{4,8,12\ldots 100\right\}$$

Additionally, the well-known Euclidean distance has been employed for calculating the cost of traveling from any client *i* to other customer *j*, using a constant 0.8, or a value 1.2 for guarantying the asymmetry feature of the problem. Furthermore, for assigning peak travel costs, an additional constant 1.2 and 1.4 (for odd or even clients, respectively) has been employed for increasing the valley travel costs.

Lastly, a pre-established amount of random street is selected as forbidden. In the following Table 1, the characteristics of each generated instances are summarized. For enhancing the understandability of this table, following clarifications should be made: *Osaba_50_1_1* and *Osaba_50_1_2* are composed by 5 clusters, which are the clusters {1, 3, 5, 7, 9}. Additionally, *Osaba_50_2_1* and *Osaba_50_2_2* are built by sets {2, 4, 6, 8, 10}. Moreover, clusters in *Osaba_50_1_3* and *Osaba_50_1_4* are comprised by 5 nodes, involving the first five customers of each cluster. On the contrary, for the construction of the 10 clusters of *Osaba_50_2_3* and *Osaba_50_2_4*, last five clients of every set are employed. Additionally, for building *Osaba_80_X* datasets first 8 clusters, or nodes (depending on the case) have been chosen. Finally, for vehicle capacities and forbidden paths, values of 240 and 5 have been chosen respectively for odd instances (*Osaba_X_X_1*, *Osaba_X_X_3* …); and values 160 and 10 for even datasets.

**Table 1:** Summary of the benchmark proposed for the AC-VRP-SPDVCFP. Forbidden paths depict the number of forbidden arcs in each cluster.

Instance	Size	Capacity	Clusters	Forbidden roads
Osaba_50_1_1	50	240	5	5
Osaba_50_1_2	50	160	5	10
Osaba_50_1_3	50	240	10	5
Osaba_50_1_4	50	160	10	10
Osaba_50_2_1	50	240	5	5
Osaba_50_2_2	50	160	5	10
Osaba_50_2_3	50	240	10	5
Osaba_50_2_4	50	160	10	10
Osaba_80_1	80	240	8	5
Osaba_80_2	80	160	8	10
Osaba_80_3	80	240	10	5
Osaba_80_4	80	160	10	10
Osaba_100_1	100	140	10	5
Osaba_100_2	100	260	10	10
Osaba_100_3	100	320	10	10
